# Potent and Quick Responses to Conspecific Faces and Snakes in the Anterior Cingulate Cortex of Monkeys

**DOI:** 10.3389/fnbeh.2020.00156

**Published:** 2020-09-29

**Authors:** Naho Konoike, Haruhiko Iwaoki, Katsuki Nakamura

**Affiliations:** Section of Cognitive Neuroscience, Primate Research Institute, Kyoto University, Inuyama, Japan

**Keywords:** non-human primate, single-unit recording, emotion, visual stimuli, neuron

## Abstract

Appropriate processing of others’ facial emotions is a fundamental ability of primates in social situations. Several moods and anxiety disorders such as depression cause a negative bias in the perception of facial emotions. Depressive patients show abnormalities of activity and gray matter volume in the perigenual portion of the anterior cingulate cortex (ACC) and an increase of activation in the amygdala. However, it is not known whether neurons in the ACC have a function in the processing of facial emotions. Furthermore, detecting predators quickly and taking avoidance behavior are important functions in a matter of life and death for wild monkeys. the existence of predators in their vicinity is life-and-death information for monkeys. In the present study, we recorded the activity of single neurons from the monkey ACC and examined the responsiveness of the ACC neurons to various visual stimuli including monkey faces, snakes, foods, and artificial objects. About one-fourth of the recorded neurons showed a significant change in activity in response to the stimuli. The ACC neurons exhibited high selectivity to certain stimuli, and more neurons exhibited the maximal response to monkey faces and snakes than to foods and objects. The responses to monkey faces and snakes were faster and stronger compared to those to foods and objects. Almost all of the neurons that responded to video stimuli responded strongly to negative facial stimuli, threats, and scream. Most of the responsive neurons were located in the cingulate gyrus or the ventral bank of the cingulate sulcus just above or anterior to the genu of the corpus callosum, that is, the perigenual portion of the ACC, which has a strong mutual connection with the amygdala. These results suggest that the perigenual portion of the ACC in addition to the amygdala processes emotional information, especially negative life-and-death information such as conspecifics’ faces and snakes.

## Introduction

The limbic system including the amygdala, cingulate cortex, and pulvinar in collaboration with the frontal cortex has been implicated in the regulation of emotional behavior (Cardinal et al., 2002; Etkin et al., 2011). The face contains information crucial for social behavior in primates (Adolphs, 2002). Thus, the quick and appropriate processing of other conspecifics’ facial information is a fundamental and essential ability both for humans and monkeys, and the limbic system is thought to be involved in the processing of such information. Single-neuron recordings and neuroimaging studies in macaques have reported that the amygdala responds to facial identity and emotions of conspecifics (Kuraoka and Nakamura, 2006, 2007; Gothard et al., 2007; Hoffman et al., 2007). In addition to the amygdala, the pulvinar and the frontal cortex also respond to conspecific faces (Tsao et al., 2008; Maior et al., 2010; Diehl and Romanski, 2014). We have previously compared how neurons in the primate amygdala and ventrolateral prefrontal cortex (VLPFC) respond to different facial stimuli (Kuraoka et al., 2015) and demonstrated that the amygdala neurons respond to the strongly negative expression of conspecifics (scream), whereas the neurons in the VLPFC prefer a facial expression with multiple meanings depending on the social context (coo). Although the cingulate cortex is one of the major components of the primate limbic system, the responsiveness of cingulate cortex neurons to conspecifics’ faces is still unclear. Neuronal tracing studies in macaque monkeys revealed that the ACC has rich neuronal connections with other limbic structures including the amygdala (Amaral and Price, 1984; Barbas and De Olmos, 1990; Kunishio and Haber, 1994; Carmichael and Price, 1995, 1996; Ghashghaei and Barbas, 2002; Kim et al., 2018), as well as other prefrontal regions (Saleem et al., 2008). Several neuroimaging studies have reported that perception of negative facial expressions modulates the neuronal activity in the ACC (Blair et al., 1999) and amygdala (Blair et al., 1999) in humans. Also, the ACC is a region critical for emotional control implicated in the pathogenesis of the psychiatric disease. Patients with depression show a reduction of gray matter volume (Drevets et al., 1997; Öngür et al., 1998; Botteron et al., 2002) and decreased activity in the subgenual portion of the ACC (Drevets et al., 1997). Furthermore, depressive patients display a negative bias in the perception of emotional stimuli such as facial expressions (Gur et al., 1992; Bouhuys et al., 1996; Hale et al., 1998). The modulation of ACC activity by negative facial expressions was more prominent in depressive patients (Gotlib et al., 2005; Chen et al., 2006). These findings suggest that the ACC functions in the processing of facial information, and that malfunction leads to the negative bias of the emotion of others in depressive patients. In addition to a conspecific’s face, the approach of a predator is also a life-and-death related emotional stimulus for monkeys, especially in the wild. Raptors, wild dogs, and snakes are potential predators for monkeys, and they elicit their fearful expression and behavior. The pulvinar neurons showed a fast and strong response to a visual image of snakes (Van Le et al., 2013). Monkeys with amygdala or anterior cingulate sulcus lesions showed a selective deficit in their emotional reactions to fake snakes (Izquierdo et al., 2005; Rudebeck et al., 2006). To understand the function of the ACC in the regulation of emotional behavior, we recorded the neuronal activity from the perigenual ACC (area 24) and examined their response to various visual stimuli including conspecifics’ faces and snakes.

## Materials and Methods

### Subjects

Two adult rhesus monkeys (*Macaca mulatta*) were used in this experiment (monkey C: 8 kg, male; monkey P: 7 kg, female). All experiments were approved by the Animal Experimentation Committee of the Primate Research Institute of Kyoto University (license No. 2012-071 and 2015-021) and were conducted following the “Guide for Care and Use of Laboratory Primates” published by the Primate Research Institute, Kyoto University (2010).

### Task Procedures

**Figure 1:**
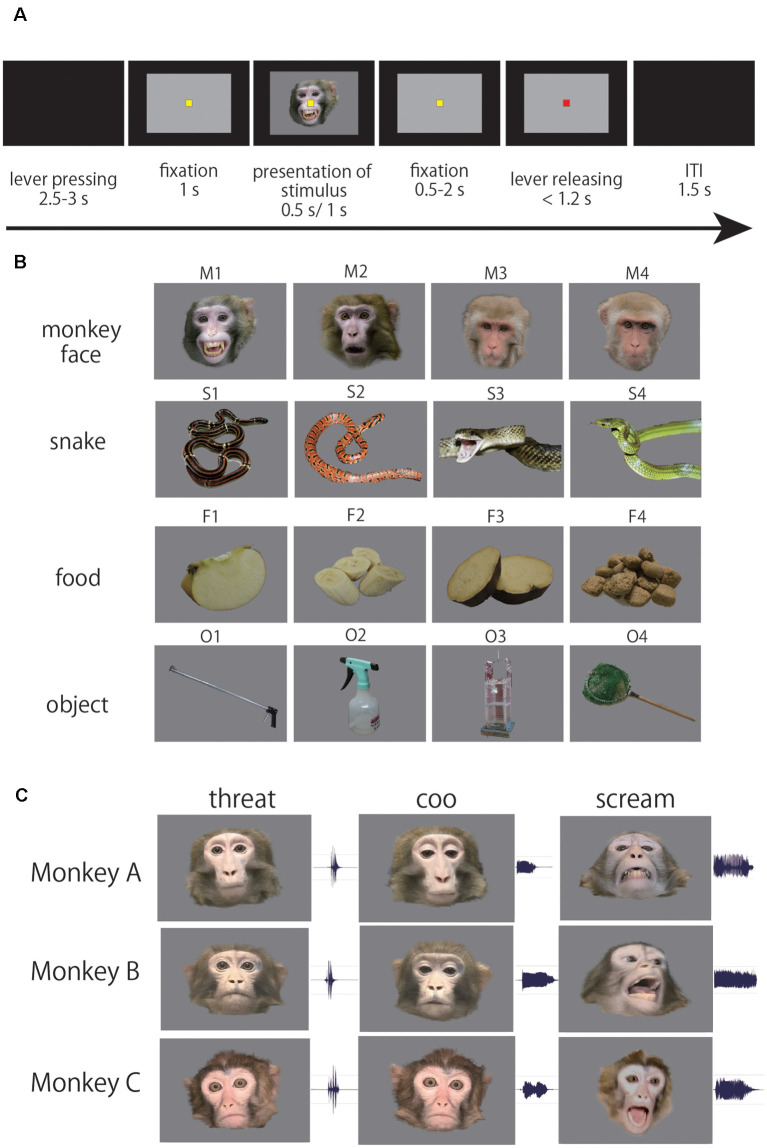
**(A)** Sequence of events in the passive viewing task. When the monkey pressed a lever, a yellow fixation point (FP) appeared. After the monkey kept the lever pressed and fixated on the FP for 1.0 s, a photo stimulus was presented for 0.5 s, or a video clip was presented for 1.0 s. Thereafter, the yellow fixation point was replaced by a red point after a certain period (0.5–2 s). If the monkey released the lever within 1.2 s of the replacement, the trial was regarded as correct. **(B)** Photo stimuli. The photo stimulus set consisted of 16 stimuli (four photos of four categories; monkey faces, snakes, foods, and artificial objects). **(C)** Video stimuli. The video stimulus set consisted of nine movie clips (three facial emotions of three monkeys; aggressive threat, coo, scream). Also, we used four videos of two human faces and two objects (camera and bell) as control stimuli (Kuraoka and Nakamura, 2006, 2007).

During the experiment, each monkey sat in a primate chair with its head fixed to the chair frame in a dark soundproof room. A lever was attached to the chair at the monkey’s waist level. At first, the monkeys were habituated to sitting in the primate chair and performed a visual reaction time task without head fixation and eye position monitoring. In this task, after the monkey pressed the lever, a yellow square appeared at the center of a monitor in front of the monkey. The monkey was required to release the lever when the color of the square changed from yellow to red. After the pre-training, for head fixation, a stainless head post was attached to the skull with stainless screws and dental-cement under sufficient anesthesia and aseptic conditions. After recovering from the surgery, the monkey was trained to perform a passive viewing task (Figure 1A). When the monkey pressed the lever and kept it pressed for a certain period (2.5–3.0 s), a yellow fixation point (FP) appeared at the center of the monitor. After the monkey kept the lever pressed and fixated on the FP for 1.0 s, a photo was presented for 0.5 s or a video clip was presented for 1.0 s. Thereafter, the yellow FP was replaced by a red point after a certain period (0.5–2.0 s). If the monkey released the lever within 1.2 s of the replacement, the trial was regarded as correct and a drop of water was delivered as a reward. If the monkey released the lever before the replacement of the FP or failed to gaze at the fixation point within 3–4°, the trial was regarded as erroneous and ended with no reward. The data from these erroneous trials were discarded. The next trial started after a 1.5 s inter-trial interval following the correct trials, and after 2.0 s following the erroneous trials.

We used two types of visual stimuli; photos and videos. To investigate what kind of information the ACC neurons process, we used the photo stimuli and compared the responses of ACC neurons to four categories of the photo stimulus. The photo stimulus set consisted of 16 pictures of four categories; monkey faces, snakes, foods, and artificial objects (Figure 1B). To examine which type of facial emotion has more impact on neuronal responses in ACC, we used video stimuli and compared the responses of ACC neurons to different types of facial emotion. The video stimulus set consisted of nine video clips of a monkey facial emotion, three monkeys (monkey A, B, C) showing three types of facial emotion (aggressive threat, coo, and scream) with their call (Figure 1C). We previously examined the responsiveness of neurons in the amygdala and ventral prefrontal cortex with the same video stimuli (Kuraoka et al., 2015). Therefore, we were able to directly compare the responsiveness of neurons among the ACC, amygdala, and ventral prefrontal cortex. Also, we used four videos of two human faces and two objects (camera and bell; Kuraoka et al., 2015). In each session, we presented the photo or video stimuli in a pseudorandom manner until the data were recorded in 10 correct trials for each stimulus. Thus, in total, the monkey completed 160 correct trials for the photo stimuli and 130 correct trials for the video stimuli.

The monitor was positioned 40 cm in front of the monkey’s eyes and these stimuli were presented on a gray background (about 30 × 20 degrees in subtended visual angle). The sounds of videos were presented at about 70 dB from speakers (SRS-Z100, SONY, Tokyo, Japan) located 55 cm in front of the monkeys. The behavioral task and the data collection were controlled by a TEMPO system (Reflecting Computing, USA), and the presentation of stimuli was controlled using custom software (written in Microsoft Visual C#). Eye positions were recorded at 30 Hz using an eye position measuring system (i_rec^1^). The monkeys performed the task for 60–120 min in each daily session. After a daily experimental session, the monkey was fed monkey chow, sweet potato, and remaining water in their home cage.

### Electrophysiological Recording

A stainless recording chamber was implanted on the monkey’s skull. For the precise placement of the recording chamber, we acquired structural MRI images (0.2T, 1.5 mm slices) of each monkey brain. We made detailed localizations of the target [the anterior cingulate cortex (ACC), Brodmann’s area 24] using the MRIcroN^2^. The chambers were implanted 31 mm and 26 mm anterior from the interaural line for the monkeys C and P, respectively. The chamber implanted on monkey P was tilted by 32 degrees, whereas that implanted on monkey C was located vertically (Figure 1C).

We extracellularly recorded the neuronal activity using tungsten electrodes (0.25 mm in diameter) with an impedance between 1 and 3 MΩ (FHC, Bowdoin, ME, USA). The electrode was lowered to the target through a guide tube (1.1 mm in diameter) with a hydraulic micromanipulator (MO-95, Narishige, Tokyo, Japan) while the monkey performed the passive viewing task.

The spike activity was monitored and converted into pulses using a multi-spike detector (ASD, Alpha Omega Engineering, Nazareth, Israel). The timing of spikes and task events were stored on an HDD of a personal computer using the TEMPO system. When the activity of a single-neuron was isolated, a recording session was started. We stored and analyzed the neuronal data only for the correct trials. We presented each visual stimulus 10 times to ensure the reproducibility and stability of neuronal responses, thus the monkey completed 160 correct trials for the photo stimuli, and 130 correct trials for the video stimuli, respectively. After we examined the responsiveness of the neuron, we advanced the electrode until the activity of the next neuron was isolated.

### Histological Analysis

**Figure 2:**
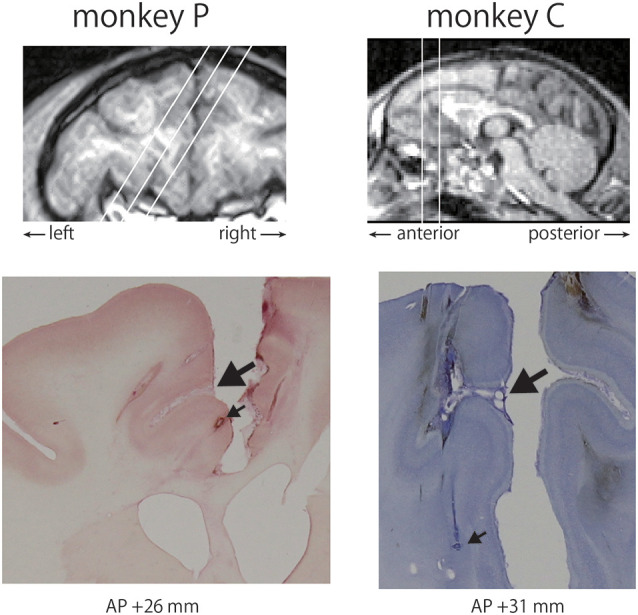
Recording sites in the anterior cingulate cortex (ACC). The chamber of monkey P was tilted by 32 degrees, whereas that of monkey C was located vertically based on T1-weighted MRI images (top). Locations of recording sites were determined using electrolytic lesions as reference points. A large arrow indicates the cingulate sulcus and a small arrow represents a lesion in each monkey (bottom).

After the final recording, small electrolytic lesions were made by passing an electric current (4 μA, 150 s using an Elgiloy electrode and a lesion maker (Digital Midgard Precision Current Source, Stoelting Co., USA) to locate the recording sites. Then, the monkeys were deeply anesthetized with an overdose of pentobarbital sodium and were perfused with 0.5 M phosphate buffer saline (PBS) followed by 8% formalin in 0.5 M PBS. The lesions were identified in 100 μm-thick coronal sections cut on a freezing microtome, and these lesions were used as reference points to determine the recording sites. Figure 2 shows Nissl-stained sections at anteroposterior (AP) + 26 mm in the left hemisphere of monkey P and AP + 31 mm in the left hemisphere of monkey C.

### Data Analysis

Neuronal data were analyzed with a custom-written script and a Statistics and Machine Learning Toolbox in MATLAB (MathWorks, Natick, MA, USA). We compared the number of spikes during the 250-ms Baseline period (749–500 ms before the stimulus onset) with that during each of the two 250-ms time windows of the Response period (1–250 and 251–500 ms after the stimulus onset) for the photo sessions, and four 250-ms time windows of the Response period (1–250, 251–500, 501–750, and 751–1000 ms after the stimulus onset) for the video sessions. If the number of spikes during either of the 250-ms time windows of the Response period was higher than that during the Baseline period (Steel’s multi comparison test, *p* < 0.05, two-tailed), the neuron was regarded as responsive to the stimulus. The optimal stimulus for each neuron was defined as a stimulus eliciting the maximal response (maximal firing rate in a 250-ms bin of the Response period) among the 16 photos or 13 video clips. The responsive neurons were divided into four groups for the photo stimuli and three groups for the video stimuli based on the optimal stimulus. For each neuron that responded to the photo stimuli, the response latency was defined as the time of the first of two consecutive 10-ms bins containing many spikes greater than the 2.33 standard deviations (i.e., *p* < 0.01) derived from the spike count in the Baseline period (Azzopardi et al., 2003; Pouget et al., 2005). To compare the mean response latency among three stimulus categories, the Kruskal-Wallis test was used.

For all 46 responsive neurons, we generated the population average of neural activity for each stimulus category. The spike density function (SDF) was generated by convolving spike times with a Gaussian filter (*σ* = 10 ms). We compared the firing rate of population activity among four categories using the Friedman test during each 50-ms period from the stimulus onset to 500 ms after the onset (total 10 bins). We plotted the Chi-square values of the Friedman test to visualize how the ACC neurons could differentiate the four stimulus categories. We also calculated the response latency for each stimulus category in the population level. First, neurons were chosen by random sampling with a replacement for each category (in the case of the “Snake” category, 16 neurons were chosen with duplicates). Then these neurons were used to calculate the mean value and standard deviation of the spike frequencies during the baseline period (−750 ms to −500 ms from the stimulus onset). We examined the time at which the SDF of the neurons in the response period first exceeded the mean spontaneous rate by 2.33 standard deviations of the Baseline period, and defined it as the response latency in the population level. These processes were repeated 1000 times by the bootstrap method and computed the 95% Confidence Intervals (CI). Data from the “Object” neurons were discarded for this analysis because the number of neurons was small (*n* = 3).

## Results

We recorded the extracellular activity from 174 single neurons from the anterior cingulate cortex (ACC) while the rhesus monkeys passively viewed the photo stimuli (133 neurons from monkey P and 41 neurons from monkey C). We also recorded the activity of 142 ACC neurons during the period when the monkeys were viewing the video stimuli (126 neurons from monkey P and 16 neurons from monkey C). The activity of 52 neurons was recorded in both photo and video sessions (43 neurons from the monkey P and nine neurons form monkey C).

### Response Characteristics of ACC Neurons to Visual Images

**Figure 3:**
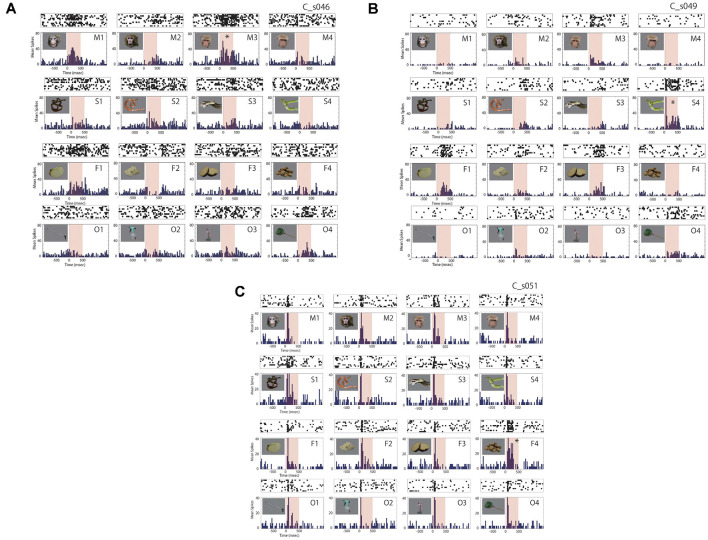
Examples of responses of anterior cingulate neurons. Raster displays and prestimulus time histograms (PSTHs) of the neural activity were aligned to the time of stimulus presentation (time 0). The neuron in panel **(A)** exhibited the maximal response to a monkey calm face (M3, marked by *) and the second-best response to a monkey grimace face (M1). The neuron also showed weaker responses to a piece of apple (F1), monkey chows (F4), a snake (S3), and a scoop net (O4). The neuron in panel **(B)** exhibited the best response to a snake (S4, marked by *) with a clear response onset after the stimulus presentation. The neurons also showed responses to a monkey calm face (M3), a piece of apple (F1), pieces of sweet potato (F3), and a scoop net (O4). It also showed weaker responses to a monkey threat face (M2), a snake (S2), another snake (S3), pieces of banana (F2), a spray (O2), but did not show any significant response to a monkey grimace face (M1), a monkey calm face (M4), a snake (S1), monkey chows (F4), a steel bar for catching (O1), or a primate chair (O3). The neuron in panel **(C)** showed responses to all stimuli immediately after stimulus onset, and show a persistent and maximal response to monkey chows (F4, marked by *).

Forty-six out of the 174 ACC neurons (26%) exhibited a significant change in firing rate in response to the photo stimuli. The ratios of the responsive neurons for the monkey P were 23% (30/133) and 39% (16/41) for the monkey C. An example of the responses of an ACC neuron is shown as averaged histograms with raster displays in the Figure 3A. This neuron exhibited the maximal response to a monkey calm face (M3, marked by *) and the second-best response to a monkey grimace face (M1). This neuron also showed weaker responses to a piece of apple (F1), monkey chows (F4), a snake (S3), and a scoop net (O4). As shown, the response onset of this neuron was not clear and was rather vague. Figure 3B shows an example of the responses of another ACC neuron. This neuron exhibited the best response to a snake (S4, marked by *) with a clear response onset after the stimulus presentation. This neuron also showed responses to a monkey calm face (M3), a piece of apple (F1), pieces of sweet potato (F3), and a scoop net (O4). It also showed weaker responses to a monkey threat face (M2), a snake (S2), another snake (S3), pieces of banana (F2), and a spray (O2), but did not show any significant response to a monkey grimace face (M1), a monkey calm face (M4), a snake (S1), monkey chows (F4), a steel bar for catching (O1), or a primate chair (O3). Most responsive neurons examined showed such stimulus preference, the ratios were 26/30 (87%) in the monkey P and 13/16 (81%) in the monkey C. On the other hand, some ACC neurons responded to all stimuli. Figure 3C shows an example of the response of such a neuron. This neuron showed clear phasic responses to all stimuli immediately after the stimulus onset. However, this neuron also showed persistent activity in response to monkey chows, the optimal stimulus (F4). This type of neurons which showed very broad selectivity and relatively shorter latency, the ratios were 4/30 (13%) in the monkey P and 3/16 (19%) in the monkey C. As shown in Figure 3, we did not find any response habituation due to the 10-times-repetitive presentation of the same stimulus.

We then examined the optimal stimulus for each responsive neuron. Of the 46 responsive neurons, 18 neurons (39%) showed the maximal response to *Monkey Face*. Sixteen neurons (35%) showed the maximal response to *Snake*. The number of neurons that exhibited the maximal response to *Food* and *Object* was 9 (20%) and 3 (7%), respectively. In the ACC, the numbers of the “*Monkey Face*” and “*Snake*” neurons were significantly larger than those of the “*Object*” neurons (chi-squared test, $\chi_{\left(3\right)}^{2}$ = 12.26, *p* < 0.01; Ryan multiple comparison test *p* = 0.002, 0.006, respectively).

**Figure 4:**
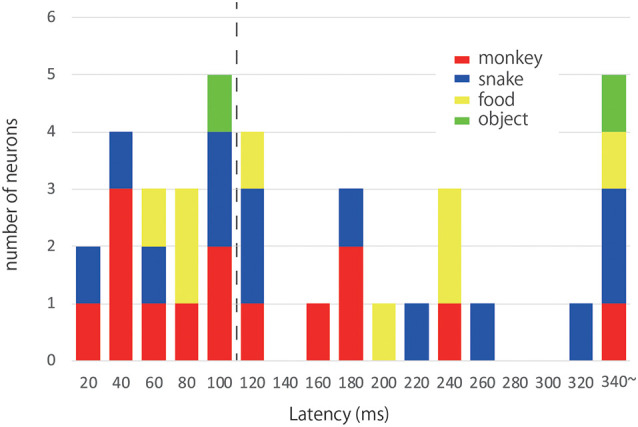
Response latencies and optimal stimuli. The histogram illustrates the distribution of latencies of the ACC neurons responsive to photos. Each color corresponds to a category of the optimal stimulus; red: *Monkey Face*, blue: *Snake*, yellow: *Food*, green: Object. The dashed line indicates the mean value of visual response latencies reported by Pouget et al. (2005). We found 18 responsive neurons with a response latency shorter than 112 ms. We defined these 18 neurons as “quick response” neurons.

We measured the latency of the maximal response of each responsive neuron (Figure 4). The median response latencies of the “*Monkey Face*,” “*Snake*,” “*Food*” and “*Object*” neurons were 127.14, 177.69, 181.25, and 245 ms, respectively. Although the median values were shorter for “*Monkey Face*” and “*Snake*” than “*Food*” neurons, there was no significant difference in the response latency between these three categories (Kruskal-Wallis test, $\chi_{\left(2\right)}^{2}$ = 0.970, *p* = 0.616). Data from the “Object” neurons were discarded for this analysis because the number of neurons was small (*n* = 3). Previously, Pouget et al. (2005) has reported that the visual response latencies in the ACC were 54–196 ms (mean 112 ± 39 ms) using a Poisson fit threshold. However, in the present study, we found 18 responsive neurons with a response latency shorter than 112 ms. We defined these 18 neurons as “quick response” neurons. As shown in Figure 4, out of 18 neurons, the optimal stimuli for nine neurons were *Monkey Face*, and those for six neurons were *Snake*.

**Figure 5:**
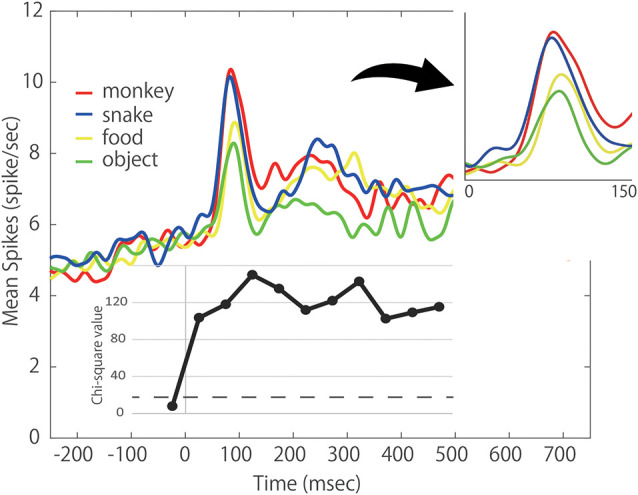
Population response of the 46 photo-responsive ACC neurons. Each colored line indicates the respective spike density function (SDF) to each stimulus category; red: *Monkey Face*, blue: *Snake*, yellow: *Food*, green: *Object*. Lower inset: chi-squared values of Friedman test comparing the firing rates between four categories during a 50 ms period just before the stimulus onset and 10 50 ms periods of the Response period. Right inset: expanded view of population activity during 150-ms after stimulus onset. All data points show a significant difference at *p* < 0.0001 level. The dashed line indicates the chi-squared value of *p* < 0.05 level.

To understand how the ACC as a whole responded to each category of stimulus, we evaluated the population average of neural activity and generated the spike density function (SDF). The SDFs of the 46 responsive neurons for four categories of stimulus are illustrated in Figure 5. As shown, the ACC neurons gradually increased their firing rate before the stimulus onset. This tendency was also observed in Figure 3A. Some ACC neurons exhibited such build-up activity preceding the stimulus presentation. However, the chi-square value in the pre-stimulus period (−50 to 0 ms before the stimulus onset) did not meet the criterion. Thus, the build-up activity did not differ between the four stimulus categories. The response consisted of the clear phasic response (the early component) and the more vague sustained response (the late component). Interestingly, the activity in response to *Snake* was higher than those to other stimuli even in the first 50 ms. As the chi-square value indicates, the SDF analysis showed that the ACC neurons started to exhibit differential responses to different categories of the stimulus within 50 ms. In the second 50 ms (51–100 ms) where the peak response of the early component was included, the responses to *Monkey Face* and *Snake* rose earlier and were stronger than those to *Food* and *Object*. In the third 50 ms (101–150 ms), the ACC neurons showed the highest ability to discriminate four stimulus categories. In this period, the ACC neurons could discriminate four stimulus categories. The response to *Monkey Face* was the strongest, that to *Snake* was the second, that to *Food* was the third, and that to *Object* was the weakest. Thereafter the late component started. The response to *Object* was always weaker throughout the Response Period while the SDFs to *Monkey Face*, *Snake*, and *Food* often crossed. Thus, the early component was stronger in response to *Monkey Face* and *Snake* than to *Food* and *Object* whereas the late component was weaker in response to *Object* than to other stimuli. In the population average level, the mean response latencies of *Monkey Face*, *Snake* and *Food* neurons were 25.93, 65.36, and 74.05 ms, respectively. The 95% CIs of these mean values to the *Monkey Face*, *Snake*, and *Food* were 23.95–27.92, 63.37–67.35, and 69.77–78.3, respectively. There was no non-overlapping of 95% CIs for the mean values among three categories. These results suggest that the response latency to *Monkey Face* was shortest, and the response latency to *Snake* was faster than that to *Food*.

All these data indicate that the ACC neurons respond more strongly and quickly to *Monkey Face* and *Snake* compared to *Food* and *Object*.

### Response Characteristics of ACC Neurons to Videos

Thirty-four out of the 142 ACC neurons (24%) exhibited a significant change in firing rate in response to the video clips of a monkey facial emotion. Of these responsive neurons, 15 neurons (44%) showed the maximal response to *Scream*, 12 neurons (35%) to *Threat*, and seven neurons (21%) to *Coo*. Thus, about 80% of the ACC neurons showed the maximal response to the negative facial stimuli, *Scream*, and *Threat*. Also, almost all (88%) of the 34 neurons responded to *Scream* and *Threat*. However, we failed to find any significant tendency that a particular type of facial emotion elicited the maximal response more frequently in the ACC (chi-squared test, $\chi_{\left(2\right)}^{2}$ = 2.883, *p* > 0.05). We also failed to find any significant difference for monkey identity in the video clips (monkey A, B, or C) elicited the maximal response in the ACC (chi-squared test, $\chi_{\left(2\right)}^{2}$ = 2.883, *p* > 0.05).

We recorded 52 neurons (43 neurons from the monkey P and nine neurons form monkey C) in both photo and video sessions. Out of 52 neurons, eight neurons (15%) showed significant activity to both photo and video stimuli (responded to at least one stimulus in each session). Also, seven neurons responded to only any of the photo stimuli, 11 neurons responded to only any of the video stimuli, and 26 neurons showed no significant change to the visual stimuli. There are no obvious differences between monkeys.

### Localization of Responsive Neurons

**Figure 6:**
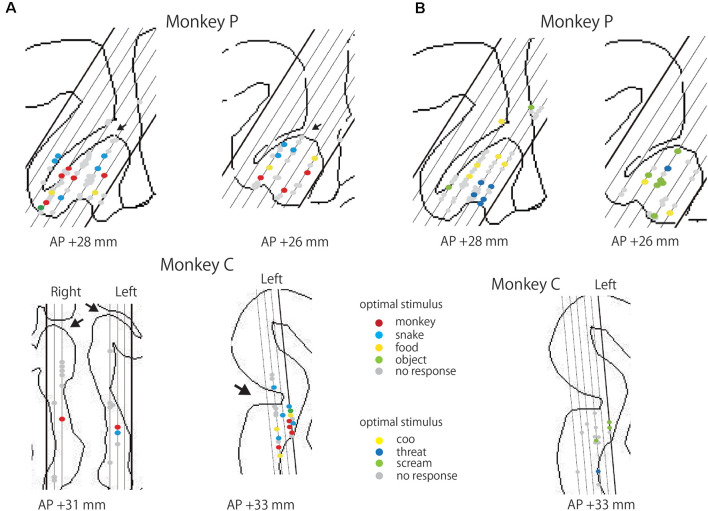
Locations of responsive neurons in the ACC. **(A)** Locations of the neurons responsive to photo stimuli from each monkey. Red, blue, yellow, and green dots indicate the neurons exhibiting the maximal response to *Monkeyface*, *Snake, Food*, and *Object*, respectively. Gray dots represent neurons that showed no significant response to any photo stimuli. An arrow indicates the cingulate sulcus. Each slanting line corresponds to each penetration in the recording chamber. **(B)** Locations of the neurons responsive to video stimuli from each monkey. Yellow, blue, and green dots indicate the neurons showing the maximal response to *Coo*, *Threat*, and *Scream* of conspecifics, respectively. Gray dots indicate neurons that showed no significant response to any video stimuli.

We examined the locations of the responsive neurons. As shown in Figure 6, for the monkey P, the responsive neurons were mainly recorded from the cingulate gyrus or the ventral bank of the cingulate sulcus adjacent to the corpus callosum. The ratios of the responsive neurons in each area were 28.6% (2/7) in the dorsal bank, 22.2% (28/126) in the ventral bank for the photo stimuli. In the monkey C, the ratios were 17.6% (3/17) in the anterior part of adjacent to the corpus callosum, 33.3% (1/3) in the dorsal bank, and 57.1% (12/21) in the ventral bank of the ACC. For the video stimuli, the ratios were 28.6% (2/7) in the dorsal bank, 25.2% (30/119) in the ventral bank in the monkey P, and 26.2% (4/15) in the dorsal bank of the ACC for the monkey C. In the monkey C, recording site was more anterior, compared to the that of the monkey P. These data suggest that the visually responsive neurons are localized in the anterior part of the ventral bank of the ACC, On the optimal stimulus, we failed to find any clusters or patches. Rather the optimal stimuli were intermingled in the ACC. Also, we found no segregated clusters based on the selectivity of responsive neurons.

## Discussion

In the current study, we examined the neuronal responsiveness to complex visual stimuli in the anterior cingulate cortex (ACC) of macaque monkeys. To our knowledge, this is the first report about the response characteristics of ACC neurons to complex visual stimuli, including videos. We found that: (1) about one-fourth of the ACC neurons responded to various visual stimuli, such as conspecific faces and snakes; and (2) monkey face or snake stimuli could elicit faster and stronger neuronal responses than other stimuli in the ACC.

We found that 26% of the ACC neurons responded to various visual stimuli. Pouget et al. (2005) used a simple figure (dotted circle) as a visual stimulus and recorded ACC neurons from two monkeys. They reported that 22% of the ACC neurons showed significant activation for the simple visual stimuli. The percentage of the responsive neurons in the ACC was similar to that of our study (26%). We found that the visually responsive neurons are localized in the anterior part of the ventral bank of the ACC, where the main recording site for monkey C. We believe that differences in the recording sites of the two monkeys induced differences in the proportion of responsive neurons for monkey C (39%) and monkey P (23%) in the photo condition.

The ACC neurons responded more to *Monkey face* and *Snake* than to *Food* and *Object* and showed a biphasic response. The early component rose up earlier and the magnitudes of the early component were larger in response to *Monkey face* and *Snake* than to *Food* and *Object*. About 80% of the ACC neurons showed the best response to *Scream* and *Threat*, which were negative facial expressions. In contrast, only 21% of neurons responded to the neutral expression, *Coo*. Thus, the ACC neurons could respond more strongly to the stimuli inducing negative emotions of conspecifics. However, we also found that some ACC neurons exhibited the maximal response to *Food* or *Object*. The ACC neurons could represent not only negative emotional stimuli but also emotionally positive or neutral stimuli. Furthermore, most of the responsive neurons responded in more than one stimulus category and showed also sensitivity to both positive and negative stimuli like Figures 3A,B (85% of the responsive neurons in monkey P and 81% in monkey C). The data suggest that the ACC neurons are considered to have a responsibility, not like so-called “grandmother cell,” but like a multidimensional response reported in other associate areas, such as the prefrontal and parietal cortex (Rigotti et al., 2013; Hadjidimitrakis et al., 2019).

Pouget et al. (2005) have examined visual response latencies in the ACC. In the present study, we found quick response neurons with shorter latency than 112 ms that Pouget et al. have reported as a mean latency of the ACC neurons. They used a simple figure as a visual stimulus, whereas we used more complex and meaningful stimuli for our monkeys. This difference in the nature of stimulus between their study and ours may explain the difference in response latency. Interestingly, in the 18 “quick response” neurons, the optimal stimuli for nine neurons were *Monkey Face*, and those for six neurons were *Snake*. The data suggests that the neurons which potentially have a strong response to Monkey face and Snakes showed a quick response to visual stimuli. Also, the medians of the response latency to *Monkey face* (127 ms) and *Snake* (177 ms) were shorter than those to *Food* (181 ms) and *Object* (245 ms). Although we failed to find any significant difference in response latency between the four stimulus categories, this is probably because of the small sample size. The difference was apparent in the SDFs. The early component is fast and phasic, and the response is similar to those in the early visual cortex. However, the ACC receives no direct input from the primary visual cortex (Vogt and Pandya, 1987). These responses may be induced by subcortical visual input including pulvinar or amygdala (Romanski et al., 1997; Pessoa and Adolphs, 2010; Kim et al., 2018).

The ACC is divided into three subdivisions: the subgenual (sgACC), pregenual (pgACC), and dorsal (dACC) divisions (Drevets and Raichle, 1998; Bush et al., 2000; Etkin et al., 2011). We identified our recording regions as area 24. Most of the responsive neurons were located in the cingulate gyrus or the ventral bank of the cingulate sulcus just above or anterior to the genu of the corpus callosum, called “pgACC” corresponding to areas 24a and 24b, respectively. The ACC has long been thought to play an important role in emotional processing (Papez, 1937). Recent studies have demonstrated that the pgACC and sgACC have distinct roles in the regulation of emotion. The reduction of resting activity in the sgACC has been observed in patients with depression (Drevets et al., 1997), whereas pgACC activity is associated with the effectiveness of antidepressants (Mayberg et al., 2005). Also, the brain areas near the sgACC and pg ACC junction shows increased activity during a variety of emotional-behavioral tasks in healthy participants (George et al., 1995; Mayberg et al., 1999; Etkin et al., 2011). In monkeys, similar functions were suggested (Hutchison et al., 2012). Results suggest that although the pgACC and sgACC have different roles, both regions may play an important role in the regulation of emotions. Negative bias could be induced when sgACC and/or pgACC activity increases or decreases and not function normally. Also, we observed build-up activity before the stimulus presentation in the ACC neurons. These data suggest that the ACC activity probably reflects not only pure visual response but also a predictive activity for an imminent emotional stimulus. Thus, the neuronal responses we found in the ACC would reflect both the visual information and expectation of the emotional content valence of the stimuli or emotional states of monkeys.

Previously, we have elucidated the functional difference between the amygdala and the ventrolateral prefrontal cortex (VLPFC). Amygdala neurons rapidly but roughly discriminated against the type of facial expressions, and preferred a fearful expression, *Scream*. By contrast, the VLPFC neurons gradually but stably discriminated against the type of expressions and preferred a communicative and multi-meaning expression, *Coo* (Kuraoka et al., 2015). Previous studies have reported that the amygdala responds strongly to negative facial expressions (Kuraoka and Nakamura, 2006, 2007; Gothard et al., 2007; Hoffman et al., 2007) and amygdala lesions could lead to fear-related emotional dysfunction such as an abnormal response of monkeys to approaching snakes (Klüver and Bucy, 1939; Aggleton and Passingham, 1982; Kalin et al., 2004; Izquierdo et al., 2005). Most of the ACC neurons which showed a significant response to video clips elicited a maximal response to *Scream* and *Threat*, which were negative facial expressions. This pattern was similar to that in the amygdala. Our present data suggest that the ACC in addition to the amygdala processes life-and-death emotional information leading to the regulation of emotional behavior.

The ACC has been reported to be responsible for decision-making based on emotional information (Botvinick, 2007). In contrast, the amygdala is thought to be involved in the processing of emotional signals (Murray, 2007; Pessoa and Adolphs, 2010). Also, neuronal tracing in macaques has revealed that the ACC has rich neuronal connections with other limbic structures, including the amygdala (Amaral and Price, 1984; Barbas and De Olmos, 1990; Kunishio and Haber, 1994; Carmichael and Price, 1995, 1996; Ghashghaei and Barbas, 2002; Kim et al., 2018). These results suggest that the ACC receives emotional information from other limbic regions and functions in a regulatory role concerning the amygdala.

Several moods and anxiety disorders such as depression cause a negative bias in the perception of facial emotions (Gur et al., 1992; Rubinow and Post, 1992; Murphy et al., 1999). Functional imaging studies have reported a decrease in the volume and activity of the ACC and an increase in activity in the amygdala in depressed patients (Drevets et al., 1997; Drevets, 2003). These previous findings together with our present results suggest that the ACC probably functions in the processing of facial emotions, and dysfunction of the neural network including the ACC and the amygdala leads to the negative bias of the perception of others’ emotions in depressive patients.

## Data Availability Statement

All datasets presented in this study are included in the article.

## Ethics Statement

The animal study was reviewed and approved by the animal welfare and animal care committee of the Primate Research Institute of Kyoto University.

## Author Contributions

NK contributed to data collection, analysis, interpretation, and wrote the initial draft of the manuscript. HI contributed to acquisition and analysis of data and assisted in the preparation of the manuscript. KN contributed to the conception and design of this study, data collection, analysis, and interpretation. All authors contributed to the article and approved the submitted version.

## Conflict of Interest

The authors declare that the research was conducted in the absence of any commercial or financial relationships that could be construed as a potential conflict of interest.

## Footnotes

^1^https://staff.aist.go.jp/k.matsuda/eye/indexj.html

^2^http://people.cas.sc.edu/rorden/mricron/main.html
